# Embedding trauma-informed, culturally sensitive, and compassionate care training in health and social care curricula: Evaluation of a multidisciplinary student intervention utilizing a pre- post-test survey design

**DOI:** 10.1371/journal.pone.0340089

**Published:** 2026-01-23

**Authors:** Frankie J. Fair, Catherine Burke, Hora Soltani

**Affiliations:** 1 College of Health, Wellbeing and Life Sciences, Sheffield Hallam University, Sheffield, United Kingdom; 2 Doncaster and Bassetlaw Teaching Hospitals NHS Foundation Trust, Doncaster, United Kingdom; National University, SUDAN

## Abstract

**Introduction:**

There were an estimated 281 million international migrants across the globe in 2020. Additionally, 30.5% of people globally have been exposed to four or more traumatic events. Providing equitable, trauma aware, culturally sensitive, and compassionate healthcare services is a priority to meet evolving health and social care requirements. This study aimed to evaluate trauma aware, culturally sensitive, and compassionate care training for health and social care students.

**Methods:**

We previously co-produced and evaluated trauma aware, culturally sensitive, and compassionate care training among midwives, with positive results across three European countries. We adapted this training for a wider range of healthcare professionals and delivered it to 1,400 students from midwifery, nursing, and other health and social care disciplines. A pre-test post-test study design using pre- and post-seminar questionnaires and a reflective paragraph submitted two months after the training evaluated the training and its impact on practice.

Descriptive statistics and Mann Whitney U-tests were undertaken on quantitative data and simple content analysis on qualitative data.

**Results:**

Significant improvements were seen between mean pre- and post-seminar scores in all domains of students’ self-perceived capability including providing culturally sensitive care (p < 0.001), trauma aware care (p < 0.001), and compassionate care (p = 0.001).

Open ended responses revealed three themes. Students identified “enhanced knowledge”, alongside “contextual influences” which acted as barriers and facilitators that impacted on “reflection and implementation in practice”.

**Conclusion:**

Training in trauma aware, culturally sensitive, and compassionate care improved students’ confidence and competence, with positive evaluations and opportunities to directly put learning into clinical practice. Incorporating such training into undergraduate curricula is essential to equip health and social care professionals for the complexities of everyday practice. Future research should place particular emphasis on evaluating students’ actual implementation of the training into practice, for example through video-taped mock clinical encounters, as well as determine the impact of training on patient clinical outcomes and experiences of care.

## Introduction

Due to political and economic instabilities, war, and sanctions, global migration is growing [1]. Currently 13% of the World Health Organization European region’s population [2] and 14.3% of the United States (US) population [3] are estimated to be international migrants or refugees. Migration influences population and socioeconomic development as well as cultural diversity. Migrants are a heterogenous group in terms of reason for migration, length of time within the host country and socioeconomic position, as well as healthcare experiences in their home country, during transit, and in the host country [1]. Migration can expose some individuals to vulnerable situations including traumatic experiences during the migration journey, communication issues, a lack of family support, and lack of familiarity with health and social care systems [4]. Migrants’ health needs are therefore complex [1], with some at higher risk of poorer outcomes, such as diabetes [5], infectious diseases [6], maternal and neonatal morbidity, and mortality [7–10].

Those from non-migrant backgrounds may also have encountered traumatic experiences, with 10.3% of United Kingdom (UK) adults and 17.3% of US adults reporting exposure to 4 or more adverse childhood events (ACEs) [11,12]. Trauma is increasingly recognized as a determinant of health, with meta-analysis suggesting those who experienced ACEs are at increased risk of numerous medical outcomes including ischemic heart disease, respiratory disease, gastrointestinal disease, memory impairment [13], and cancer [14]. ACEs are also linked to adverse psychosocial and behavioral outcomes such as tobacco use, alcohol problems, depression, substance misuse, obesity, suicidal ideation, and anxiety [13]. Trauma can also impact upon interpersonal skills and problem-solving ability [15]. It can also lead to hypervigilance and difficulties with emotional regulation when the trauma response is triggered leading to fight, flight, freeze responses manifested through behaviors such as aggression and withdrawal [15].

When considering trauma more widely, not just within childhood, a worldwide survey suggested that 30.5% of people had been exposed to four or more traumatic events [16]. It is therefore essential that services provide trauma-informed care to avoid re-traumatizing individuals exposed to previous trauma [15]. A trauma informed service recognizes the widespread impact of trauma, understands the signs of previous trauma and responds through integrated policies, procedures, and practices while actively resisting re-traumatization [17].

Health service provision that is equitable, compassionate, trauma aware, and culturally sensitive is required to meet the needs of diverse communities [18]. As part of a collaborative and interdisciplinary international project, to reduce health disparities for pregnant migrant women from ethnic minority backgrounds (https://oramma.eu/the-project), we previously: 1. Systematically synthesized evidence about migrant women’s needs and experiences of maternity care [4]. This highlighted that migrant women face specific psychosocial and economic challenges which require cross-agency working and trauma-informed models of care, as well as the provision of social and emotional support. 2. Developed trauma aware, culturally sensitive, and compassionate care training materials for healthcare providers. 3. Implemented and evaluated the training among midwives in three European settings with different sociocultural and health systems [19]. This training showed positive results and evidence of impact [19]. Cultural competence training for undergraduate students has previously been identified as lacking for professions such as student nurses both within the UK [20] and other European counties [21], as well as in a global review which found no previous studies of trauma aware education for student nurses [22]. The importance of providing such training to a wider range of health and social care students was therefore recognized as an important step to upskill the workforce given the limited previous content regarding this within the curriculum.

## Methods

### Training development

Within the host university health and social care students participate in an integrated curriculum, with aspects of interdisciplinary shared learning. Our previous training was adapted for students within this integrated care curriculum including midwives, nurses, physiotherapists, operating theatre practitioners, occupational therapists, paramedics, and radiographers.

Adaptation of the training materials occurred through discussion with a multi-disciplinary team of health and social care professionals, and a psychologist. The content included benefits and challenges associated with cultural diversity and related health needs, cultural awareness exercises with individual reflection on power/privilege, the potential causes and impact of trauma, the concept of compassionate care rather than pity or sympathy, and the importance of self-care. A ‘talking head’ from a service user with lived experience of trauma and mental health was also included, with an opportunity for students to ask the service user additional questions about their background, illness, and interaction with professional services. The service user recorded feedback in response to student’s questions. The initial seminar day included 5 hours of content, with an additional hour of self-directed learning content.

The trauma aware, culturally sensitive, compassionate care training was delivered to students through ‘training the trainers approach’, with a half day training session for staff who would facilitate the multi-disciplinary student seminar groups. These facilitators then rolled out the content to approximately 1400 second year students enrolled on courses within the integrated curriculum.

### Study design

A pre- and post-test survey methodology was adopted, with students completing a survey prior to the seminar and another after attending the seminar. Self-perceived capability to provide trauma aware, culturally sensitive, and compassionate care was assessed on a 0–10 scale, with 10 being completely competent. The Cultural Competence Groningen Reflection Ability Scale (CC-GRAS) was used to assess personal reflection ability [19,23]. Additional Likert scale questions were included to agree or disagree with statements relating to compassion and compassionate care, as used within previous research [24,25]. Within the post-seminar survey additional open-ended questions collected students’ views of seminar participation. Questionnaire data was collected between 27^th^ September 2022 and 4^th^ November 2022.

Two months after the seminar, after students had returned to a practice-based environment, they were offered the opportunity to submit a reflective paragraph to describe how they had implemented into practice the knowledge learnt within the seminar. These paragraphs were to be submitted by 20^th^ December 2022. Students were advised a £10 gift voucher would be given to the ten best reflective paragraphs.

### Data analysis

Data analysis was performed using SPSS version 26.0. Descriptive statistics summarized the students’ characteristics and average responses to each question. The overall CC-GRAS score was only calculated if no more than 2 sub-domain items were missing. Data was not matched, with differences in pre-seminar and post-seminar scores analyzed using the Mann Whitney U-test as data was not normally distributed.

Responses to open ended survey questions and reflective paragraphs were analyzed using simple content analysis. Content analysis is a systematic approach to categorize large quantities of textual data [26,27]. Inductive content analysis was used, given the lack of previous study within this area [26,27]. The open-ended responses were initially read for familiarity. After this, responses were coded independently by 2 researchers with these codes derived directly from the data to capture key thoughts or concepts [26.27]. A code was identified for each open-ended response, with more than one code identified for many responses. Through discussion between the researchers, these codes were then grouped into categories of related codes [26]. Categories were grouped into themes and subthemes, with consideration given for how these themes inter-related [27]. Final themes and subthemes were agreed by all authors. Examples for each subtheme were identified within the data to illustrate each subtheme within the analysis [26,27].

### Ethical considerations

Ethical approval was obtained for this project Sheffield Hallam University (Converis Number ER45054854) on 12/08/2022. Researchers were not involved in seminar delivery. Students were directed to the survey within online course materials and by seminar facilitators. Students were provided with information regarding the survey’s purpose, including a statement that those who wanted to participate could complete the electronic survey. Therefore, completing the survey was considered as giving written consent. Voluntarily returning practice related feedback to the researchers was also taken as inherent consent. No identifiable information has been reported to protect student confidentiality. When reporting quotations the ethnic group of each student has been categorized broadly as White British, White other, Asian, Black, or Mixed to maintain confidentiality.

## Results

### Quantitative results

At least one survey response was provided by 620 students. Table 1 shows a summary of the students’ characteristics. A total of 185 students provided both pre- and post-seminar responses, 332 just completed the pre-seminar survey, and 103 just completed the post-seminar survey. Over 25% of the students were from a non-White British background. Only 71% of students reported that they and both of their parents were born in the UK.

**Table 1:** Student characteristics.

Characteristic	Overall (n = 620)
**Age**	26.0 (±8.5) years Range 19–58 years (n = 590)
**Gender** Female Male Prefer not to say NR	496 (80.0%) 100 (16.1%) 8 (1.3%) 16 (2.6%)
**Ethnicity** White British White Irish Other White, e.g., European/ South African Black African Black Caribbean Black – other Asian Bangladeshi Asian Chinese Asian Pakistani Asian Indian Asian other Arab Mixed – White and Asian Mixed – White and Black Caribbean Mixed – White and Black African Mixed – other	456 (73.5%) 5 (0.8%) 13 (2.1%) 44 (7.1%) 7 (1.1%) 2 (0.3%) 5 (0.8%) 8 (1.3%) 35 (5.6%) 5 (0.8%) 10 (1.6%) 6 (1.0%) 2 (0.3%) 11 (1.8%) 5 (0.8%) 6 (1.0%)
**Migrant status of student** Born in UK and both parents born in UK Born in UK but one/both parents born outside UK Born outside of the UK and one/both parents born outside of UK Born outside of UK but parents born in UK NR	440 (71.0%) 69 (11.2%) 80 (12.9%) 6 (1.0%) 25 (4.0%)

NR = not reported; UK = United Kingdom.

Significant improvements between mean pre- and post-seminar scores were seen in all domains of students’ self-perceived capability (see Table 2), including to provide culturally sensitive care (p < 0.001), trauma aware care (p < 0.001), and compassionate care (p = 0.001). Pre- and post-test scores were assessed by ethnicity (white British vs any other ethnicity) and migrant status (non-migrant background vs first or second-generation migrant) and are given in S1 Table. Significant improvements were seen in self-perceived capability to provide culturally sensitive care and trauma aware care regardless of ethnicity or migrant status. Although improvements were seen between mean pre-test and post-test scores for self-perceived capability to provide compassionate care in all subgroups, this was not significant for those from a non-White British ethnicity or among those with a first generation or second-generation migrant background.

**Table 2:** Students mean self-perceived capability in each domain before and after the training seminar.

	Pre-test			Post-test			
Question	Mean	SD	N	Mean	SD	N	P value
To what extent do you feel capable to provide culturally sensitive care (care that is sensitive to people of a different culture to you)	7.08	1.87	516	8.31	1.33	288	<0.001
To what extent do you feel you are capable to provide trauma aware care (having an awareness of the different types of trauma people may have experienced and how that may impact care)?	6.44	1.87	517	8.07	1.39	286	<0.001
To what extent do you feel capable to provide compassionate care?	8.56	1.36	516	8.92	1.07	288	0.001
CC-GRAS total score	37.73*	5.16	516	40.62‡	4.53	288	<0.001

SD – standard deviation.

CC-GRAS – Cultural Competence Groningen Reflection Ability Scale.

* 12 cases had one sub-component score missing (2.3%), 2 cases had 2 missing scores (0.4%) and one case was removed as there were more than 2 sub-component scores missing.

‡ 6 cases had 1 missing sub-component score (2.1%), 2 cases had 2 missing scores (0.7%) and no cases had more than two missing scores so no cases were removed from the analysis.

The CC-GRAS mean score increased from 37.7 pre-seminar to 40.6 post-seminar (p < 0.001) (Table 2). Individual pre- and post-seminar scores for the ten subcomponents within the CC-GRAS are given in S2 Table. All except one subcomponent had significantly higher mean post-seminar score than pre-seminar score.

After the seminar students felt more capable of handling cultural differences (p < 0.001), of communicating sensitively (p < 0.001), and of managing cases where there was a language barrier (p < 0.001) (Table 3). They also reported a clearer understanding of what trauma aware care (p < 0.001), and compassionate care (p < 0.001) involved.

**Table 3:** Student’s understanding of cultural sensitivity, trauma aware care and compassion.

	Pre-test			Post-test			
Question	Mean	SD	N	Mean	SD	N	P value
I feel capable of: • Communicating in cases when there is a language barrier • Handling cultural differences • Communicating sensitively	3.13 3.60 4.12	0.905 0.827 0.699	515 514 516	3.71 4.06 4.38	0.858 0.741 0.618	287 287 287	<0.001 <0.001 <0.001
I have a clear understanding of what trauma aware care is	3.14	0.872	515	4.32	0.691	286	<0.001
I have a clear understanding of what compassionate care is	4.34	0.700	515	4.62	0.534	288	<0.001
Compassion is difficult to define but you know if it is there or not	4.14	0.838	513	4.42	0.723	288	<0.001
Compassion is something that can be taught and learnt	3.37	1.034	513	3.67	1.069	285	<0.001
Compassion is something that can be measured	2.98	1.027	515	3.33	1.239	284	<0.001
In order to provide compassionate care, health and social care professionals need training	3.64	1.037	513	3.84	1.046	287	0.004

SD = standard deviation.

The majority of students (n = 270, 94%) completing the post-seminar survey felt they would have an opportunity to put what they had learnt during the seminars into practice. Sixteen students (5%) were unsure whether they would have an opportunity to put their learning into practice, with two students (<1%) feeling they wouldn’t have such an opportunity.

### Open-ended survey responses

**Fig 1:**
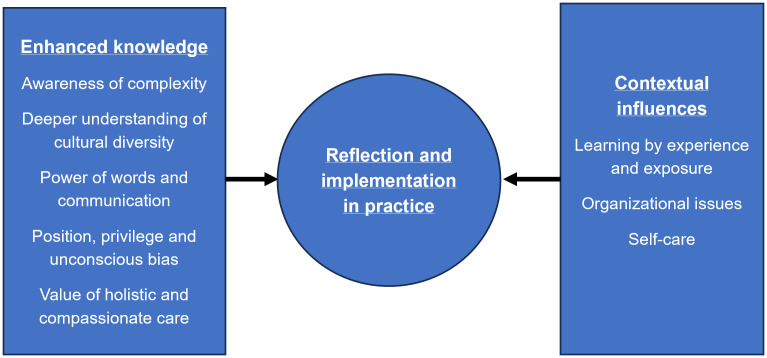
Open ended survey response themes.

Three themes were generated from open-ended survey responses. These are illustrated, alongside their subthemes in Fig 1 and discussed below. Illustrative quotations are provided below and in S3 Table.

### Enhanced knowledge

#### Awareness of complexity.

When asked what they had learnt during the seminar and could implement in practice, the trauma component of the training was the most commonly mentioned, with 44.6% of students (104 of 233 responses) specifically mentioning this. Students particularly appreciated learning about the impact of trauma on health, the potential influence of trauma on a person’s behavior such as distancing themselves or becoming emotionally distressed, recognizing that situations may be triggering including in situations where an individual’s trauma history is unknown and the importance of focusing on survivors’ resilience rather than seeing them as victims. Students became more aware of the complexity and individuality of every situation as they recognized the impact on care of clients from different ethnicities, circumstances, and trauma backgrounds. Students also realized their colleagues may have their own trauma backgrounds, that may need to be understood and managed.

*“Remember that everyone may or may not be struggling with something.”* [Female, White Other, Child Nursing; line 132]

*“Some people’s trauma may be obvious, but other’s may have hidden trauma which affects their behavior; therefore we should always be careful especially when providing personal care.”* [Female, White British, Adult Nursing; line 85]

“*How to address individuals in a more conscientious way if they have faced a traumatic event.*” [Female, Asian, Occupational Therapist; line 35]

#### Deeper understanding of cultural diversity.

Students demonstrated a deeper understanding of ethnic inequalities within healthcare and respect for cultural diversity after the seminar. They appreciated the opportunity to consider other cultures and the reasons for and necessity of migration to the economy.

*“There is no better or bad culture but they are different.”* [Male, Black, Diagnostic Radiography; line 222].

*“Migration and diversity are essential for survival and growth.”* [Female, White British, Social Work; line 109]

#### Power of words and communication.

Communication was an important subtheme described by students. They had an increased understanding of the power of their own words to escalate or de-escalate situations, as well as to alienate or build trust. Students described learning about the importance of actively listening and validating the service user, as well as how to recognize non-verbal communication, especially to observe when others may be feeling uncomfortable.

*“Words are so powerful. Be wise on the words used in any situation and make sure to think ahead before speaking.”* [Female, Asian, Physiotherapy; line 141]

*“To be more aware of how I word things and the effects they may have on service users.”* [Female, White British, Midwifery; line 155]

#### Position, privilege and unconscious bias.

Students reported becoming more aware of their unconscious biases and the privilege and power accorded by their position as a healthcare professional. They recognized that to achieve equity within care that healthcare providers need to be self-aware, emotionally intelligent, reflective, and non-judgmental. Students described learning to remain more open-minded and respectful within every interaction.

*“The privilege scale really shows how society reacts to different people even if you may not notice it.”* [Female, White British, Mental Health Nursing; line 96]

*“We naturally have an unconscious bias, and this influences our care delivery and service users’ experience in and outside of practice. However, it is possible to change my unconscious bias.”* [Female, Mixed ethnicity, Adult Nursing; line 208]

#### Value of holistic and compassionate care.

After the seminar students were more aware of the need to provide holistic, person-centered care, recognizing the service user as the expert. Students’ confidence to approach situations with sensitivity and empathy therefore increased. Students recognized they were responsible for forming trusting relationships and creating opportunities for service users to disclose any concerns or needs.

*“To be person centered with every individual.”* [Female, Black, Adult Nursing; line 157].

*“Creating opportunities for people to feel safe & secure.”* [Female, White British, Child Nursing; line 161]

### Contextual influences

When asked what might prevent them putting what they had learnt during the seminar into practice, 19 students felt there would be no barriers. Other students reported numerous potential barriers as well as identifying factors that may facilitate implementing trauma aware, culturally safe, and compassionate practice. These are described below.

#### Learning by experience and exposure.

Many students felt a lack of experience and knowledge was a barrier to implementation and they were worried about ‘getting it wrong’. The complexity and individuality of every situation added to this lack of confidence. Several reported that ‘only being a student’ meant their concerns or ideas on placements were not taken seriously and meant they felt underconfident to challenge other’s practice.

“*Having the confidence to challenge or discuss your views with an experienced practitioner can be difficult*.” [Female, White British, Midwifery; Line 122]

Despite the training aiming to raise awareness of trauma and different cultures, five students reported a barrier to implementing their learning into practice as a lack of contact with people who had experienced any trauma.

“*Being in a placement area that doesn’t have patients with these issues.”* [Female, White British, Adult Nursing; line 163]

### Organizational issues

Many organizational issues were cited as barriers to implementation. These included the workplace culture, including qualified staff’s lack of awareness, and current UK health funding structures being linked to specific targets that don’t include compassionate care. Students felt respect for service users and patient-centered care were lacking in their practice placements, which resulted in poor care plans. A resistance to change within organizations was also noted.

*“Other individuals not having the awareness of what compassionate and trauma aware care entails*.” [Female, White British, Child Nursing; line 62]

*“Too much focus on ticking boxes for placement rather than focus on the patient themselves.”* [Female, White British, Adult Nursing; line 68]

While unrushed communication and actively listening was seen as essential, the difficulties of achieving this in practice where interactions are time pressured was noted by numerous students. Poor communication due to language barriers and inadequate interpreter services were also noted as a barrier to building trusting relationships and providing patient-centered care.

*“Time constraints, however, this is no excuse. I’m going to make time.”* [Female, White British, Adult Nursing; line 22].

*“[I] Have previously found it hard to use translation services.”* [Female, White British, Midwifery; line 128]

### Self-care

Several students noted they themselves could be a barrier to implementation due to the emotional demands on them, for example due to their own ACEs, current difficult family situations or the reality of daily life. Students highlighted the self-care content of the training, recognizing proactive self-care would enable them to deliver trauma aware, culturally sensitive, and compassionate care.

*“Health care professionals are likely to have ACE’s themselves.”* [Female, White British, Child Nursing; line 216]

*“Personal issues such as home dynamics on a daily basis. For example if things are well organized for the day and in place [childcare, finances, relationship with spouse, siblings, and immediate family members etc] you are rest assured you will be in good spirit when working in practice.”* [Female, Black, Adult Nursing; line 131]

### Reflection and implementation in practice

#### About the course.

Most students found the new course content informative, interesting, eye-opening, relevant, and enjoyable.

*“I really enjoyed today’s session and it opened my mind to new things I wasn’t even aware of before.”* [Female, White British, Adult Nursing; line 5]

*“Very interesting and thought-provoking topic.”* [Male, White British, Adult Nursing; line 218]

However, two students felt that becoming culturally competent and trauma informed takes years of practice and another student felt it was just common sense.

*“To be ‘truly trauma aware & culturally sensitive’ takes years of experience working with and dealing with a wide range of individuals from all cultures and backgrounds. It can’t be taught in a classroom.”* [Male, White British, Physiotherapy; line 159]

Students enjoyed the learning style, especially the talking head, the opportunity for group discussions and the walk in the park exercise that highlighted areas of unconscious bias.

*“Hearing [talking head’s name] story and how she has been poorly treated by professionals in the past.”* was deemed as the most important part of the seminar by one student. [Female, White British, Adult Nursing; line 22]

Most students didn’t have any ideas on how to improve the seminar. However, some wanted more talking heads and for multiple shorter sessions rather a full day seminar.

### Assessing longer term implementation impact

Students were encouraged to submit a short reflective paragraph two months after the seminar to assess the longer-term impact. Ten students provided a paragraph. They described enhanced self-awareness around unconscious bias and increased understanding of providing trauma aware, cultural sensitivity, and compassionate care in practice. The paragraphs demonstrated students’ ability to reflect on practice-based situations improved after the seminar and their success in implementing what they had learnt when caring for service users with previous trauma. Illustrative quotations from the reflective paragraphs are given in Table 4.

**Table 4:** Illustrative quotations from student reflective paragraphs.

*“During placement, I encountered a mentally unwell patient who had full mental capacity. The patient refused to be admitted to the hospital despite numerous attempts from the crew, which led to the crew becoming more frustrated.* … *Negative stereotypes can cause healthcare professionals to communicate less effectively, leading to the patient feeling less comfortable about explaining their condition. … In order to ensure a more patient-centred approach in future, I will continue to write frequent reflections after jobs where patient outcomes were not met, challenging my biases and uplifting my beliefs around said patient.” [Paramedic]*
*“I went to visit an individual in hospital who had suffered different events such as physical abuse and a loss of her own baby … Before the assessment I spent a few minutes thinking about how to speak to this individual about her feelings and past traumas in a way that was sensitive. On approaching the patient, we assured her that this is a safe space to speak … I ensured to recognise my unconscious bias and refrain from making any judgements.... The individual got increasingly upset within the assessment, so I tried to use empathy to reassure the patient. We did end up discussing parts of the individuals’ trauma, but this was led by the patient and we found that she had not received any sort of counselling for these traumas so we were able to complete a referral for this patient.”* [Mental Health Nurse]
*“I found the “Walk in the Park” exercise particularly insightful during this lecture to highlight the subject of unconscious bias and how this impacts our thoughts, feelings, and behaviours in every day scenarios. … Although recognising, understanding, and addressing my own biases feels uncomfortable at times, it is a vital journey to follow to improve everyday practice, making me a more inclusive, authentic practitioner.”* [Midwifery]
*“Throughout my transition into healthcare, I have thought a lot about three feelings (hopelessness, loneliness, and guilt) which are inherent to depression but are also innate in all of us. I believe feelings of loneliness and guilt, which any patient may be experiencing, are particularly under-supported in healthcare and I feel it is important for us to recognise the cultural biases which may influence them.”* [Diagnostic Radiography]
*“I have learnt from my placement that person-centred care is the most important role of being a nurse or a support worker … Showing compassion, empathy, and courage towards a patient and showing that you’re there for them and you’re showing them that they are an individual and not like everyone else.”* [Adult nurse]

## Discussion

Our previously developed trauma aware, culturally sensitive, and compassionate care training was effective among midwives from three different European countries [19]. This current evaluation also demonstrated a clear value to delivering this trauma aware, culturally sensitive, and compassionate care training to students across a multitude of healthcare disciplines. Open ended comments showed the training was well received, with quantitative evaluation also indicating enhanced self-perceived capability to provide trauma aware, culturally sensitive, and compassionate care.

This training clearly addresses the need identified within numerous previous studies for more training on culturally competent care for healthcare professionals. For example, previously no specific focus on cultural competency was found within the nursing curriculum in any of the four European countries investigated [21] and none of the UK student nurses’ interviewed in another study received any formal cultural competence training within their course [20]. This lack of formal cultural competence training was a concern to the students [20], with students considering culturally competent training to be important [21]. When provided with the opportunity to learn about cultural sensitivity, and consider how their unconscious biases may impact the care they provide, students within this current research were overwhelmingly positive. While becoming culturally competent is recognized as an ongoing process, which is why the term cultural sensitivity was preferred within this research, this research as well as a previous evaluation of cultural care training for student nurses found significant improvements in cultural competence [28]. Student nurses within the previous evaluation received five 2-hour long sessions [28], while this current training was delivered in a one day seminar. As both showed significant improvements in cultural competence, it suggests any training may beneficially improve healthcare student confidence and competence and provide an opportunity to enhance healthcare professionals’ cultural competence [20].

Gaps have also previously been identified in trauma awareness training for healthcare professionals and healthcare students. A review of trauma-informed education in health concluded that more integration of trauma informed care into nursing education programs is required [22]. They found no articles of trauma informed education for student nurses despite being present in other health related disciplines such as dentistry, medicine, and social work [22]. This lack of trauma specific training is despite the widespread prevalence of trauma within society [29]. The need for self-awareness when responding to trauma is essential, as well as an understanding of vicarious trauma, where the caregiver themselves can experience trauma from repeatedly hearing others trauma narratives [29,30]. This is especially important given the increasing recognition of burnout amongst professionals [30], and the large number of nurses and midwives subsequently leaving the profession [29]. There is also increasing recognition that a high proportion of those entering healthcare professions have themselves experienced trauma. One study found 31.7% of student nurses had experienced 4 or more ACEs, far higher than in the general adult population [31]. While some students felt their childhood experiences were beneficial making them more emotionally aware within their practice [31], the high prevalence of ACEs amongst healthcare providers highlights the critical importance of teaching trauma informed care to healthcare students. This is essential to equip students to care for those who have experienced trauma, to ensure their own long-term ability to care and therefore enable them to remain in the profession [30].

The positive improvements in self-perceived confidence to provide trauma aware care within this current study is comparable to previous research of trauma aware training among healthcare professionals. This has suggested trauma awareness training can enhance staff knowledge, confidence, attitudes, and behaviors [15,32,33], including up to 1 month after the training [32]. While observing the impact of the current training on actual practice or on client related outcomes was outside the realm of this study, previous literature has demonstrated that improved knowledge and confidence from trauma informed training are likely to enhance clinical practice and service user outcomes [32,33], although this is not unanimous with one review finding many staff felt they lacked the skills required to actually implement trauma informed care into practice [15]. Incorporating service users into trauma informed care development has particularly been noted to enhance the success of implementation previously [15]. Within this current training the client talking head was similarly well received, triggering a plethora of further questions from the students, as well as being highly evaluated.

The concerns raised by students about the potential barriers for implementing what they had learnt into practice resonate with the previous literature. Many have previously noted language to be a key barrier for people from diverse backgrounds [20,21], especially given the lack of interpretation facilities to enable effective communication [20,34]. These language barriers result in inadequate care, lack of access to care and a lack of trusting relationships [21]. The lack of integration of cultural assessments into care plans has also previously been identified by student nurses as a barrier to culturally competent care [20]. Organizational issues such as time constraints [15], staff shortages [15,20,34], rigid policies and procedures [15,34], lack of awareness at a managerial level [34], and staff resistance to change [15] are also all reported within the literature as barriers to implementing person-centered care. While most previous research on implementation of trauma informed care has been undertaken in the United States and occurred within mental health settings [15], clear parallels to the perceived barriers can be seen within this research with a range of healthcare professional students.

### Study strengths and limitations

This study evaluated training implementation within a large cohort of students across a wide range of health and social care disciplines, which is seen as a strength. Additionally, the evaluation incorporated previously used scales including the CC-GRAS [23] and attitudinal statements relating to compassion and compassionate care [24,25].

However numerous limitations were noted. The study was undertaken in a single university. Face-to-face training of all seminar facilitators was challenging, however recording the ‘train the trainers’ event ensured all facilitators could access the training content. Resources used were consistent across all student seminar groups, however it was not possible to evaluate actual content delivery to ensure consistency. Not all students completed the pre- and post-seminar surveys. Facilitators who prompted students to complete the survey may have been those who recognized the topic’s importance and so facilitated the seminar in a more confident and engaging way. Additionally, students who completed the post-seminar survey may have had more positive views, resulting in selection bias. However, the proportion of survey respondents who were male (16.1%), over 21 at course commencement (50.8%) or from Black, Asian or minority ethnic communities (23.4%) corresponded closely with the profile of students enrolled on these courses being 16.3%, 52.7% and 22.2% respectively, suggesting a fairly representative sample. This study did not include a control group which might have weakened external validity. However, pre-, post-test study designs are widely used and considered to have adequate reliability to assess training effectiveness [35].

While reflective paragraphs evaluated the long-term impact of the training, it was not possible to observe actual impact on practice. Future evaluation could observe students’ actual implementation in practice for example through video-taped mock clinical encounters, as well as assessing whether changes in practice are sustained over longer periods of time.

### Implications

This research showed that incorporating trauma aware, culturally sensitive, and compassionate care training into the undergraduate curricula for health and social care students was effective at increasing student’s self-perceived competence. Given increases in global migration noted over recent years and increased recognition of trauma as a determinant of health, incorporating such training into the undergraduate curriculum is deemed essential to equip new health and social care professionals for the complexities they will face in everyday practice.

The ‘train the trainers approach’ utilized within this study to assist with large scale roll out of the new curricula proved effective at equipping staff who would deliver the content and decreased staff resistance to teaching the new material. However, in the future due to staff turnover, annual leave and sickness, more than one ‘train the trainers’ event would be advisable to ensure all staff timetabled to deliver the new content had the opportunity to attend.

It is essential that any future research in this area place a particular emphasis on examining actual changes in students’ behavior through direct observation or determining the impact of the training on patient care both on clinical outcomes and patient experiences of care. Longer term follow-up would also be beneficial to determine whether changes in attitude and behavior are sustained.

## Conclusion

After delivering trauma aware, culturally sensitive, and compassionate care training seminars, students showed improved self-perceived capability to deliver care in all areas. Training was positively evaluated by students, with opportunities identified to directly put learning into clinical practice and an impact narrated within students’ qualitative reflective paragraphs.

## Supporting information

S1 Table Pre- and post-seminar scores according to student ethnicity and migrant status. (PDF)

S2 Table Pre and post-seminar scores for each sub-component of the CC-GRAS score. (PDF)

S3 Table Additional quotations. (PDF)
