# Supplementary material for: Embedding trauma-informed, culturally sensitive, and compassionate care training in health and social care curricula: Evaluation of a multidisciplinary student intervention utilizing a pre- post-test survey design
Source: PLoS One. 2026 Jan 23;21(1):e0340089. doi: 10.1371/journal.pone.0340089 (PMC12829834; doi:10.1371/journal.pone.0340089)
Supplement: S1 Table — (PDF) [file pone.0340089.s001.pdf]

## S1 Table. Pre- and post-seminar scores according to student ethnicity and migrant status

| | Pre-test | | | Post-test | | | |
|---|---|---|---|---|---|---|---|
| **Subgroup analysis for each question** | **Mean** | **SD** | **N** | **Mean** | **SD** | **N** | **P value** |
| **To what extent do you feel capable to provide culturally sensitive care (care that is sensitive to people of a different culture to you)** | | | | | | | |
| White British | 7.00 | 1.82 | 395 | 8.35 | 1.29 | 200 | <0.001 |
| Any other ethnicity | 7.36 | 2.03 | 121 | 8.22 | 1.41 | 88 | 0.003 |
| Non migrant background | 7.01 | 1.85 | 386 | 8.34 | 1.30 | 190 | <0.001 |
| First or second generation migrant | 7.35 | 1.96 | 123 | 8.19 | 1.45 | 81 | 0.002 |
| **To what extent do you feel you are capable to provide trauma aware care (having an awareness of the different types of trauma people may have experienced and how that may impact care)?** | | | | | | | |
| White British | 6.49 | 1.80 | 395 | 8.25 | 1.25 | 199 | <0.001 |
| Any other ethnicity | 6.27 | 2.05 | 122 | 7.67 | 1.60 | 87 | <0.001 |
| Non migrant background | 6.48 | 1.82 | 386 | 8.22 | 1.26 | 190 | <0.001 |
| First or second generation migrant | 6.33 | 2.00 | 124 | 7.66 | 1.68 | 80 | <0.001 |
| **To what extent do you feel capable to provide compassionate care?** | | | | | | | |
| White British | 8.60 | 1.31 | 395 | 8.97 | 1.08 | 200 | 0.001 |
| Any other ethnicity | 8.44 | 1.52 | 121 | 8.82 | 1.06 | 88 | 0.218 |
| Non migrant background | 8.61 | 1.30 | 386 | 8.96 | 1.09 | 190 | 0.002 |
| First or second generation migrant | 8.45 | 1.47 | 123 | 8.80 | 1.05 | 81 | 0.226 |
| **CC-GRAS total score** | | | | | | | |
| White British | 37.36 | 5.15 | 394 | 40.74 | 4.61 | 200 | <0.001 |
| Any other ethnicity | 38.93 | 5.02 | 122 | 40.34 | 4.35 | 88 | 0.033 |
| Non migrant background | 37.31 | 5.22 | 386 | 40.69 | 4.69 | 190 | <0.001 |
| First or second generation migrant | 39.10 | 4.77 | 124 | 40.35 | 4.33 | 81 | 0.048 |

SD – standard deviation
