# Supplementary material for: Embedding trauma-informed, culturally sensitive, and compassionate care training in health and social care curricula: Evaluation of a multidisciplinary student intervention utilizing a pre- post-test survey design
Source: PLoS One. 2026 Jan 23;21(1):e0340089. doi: 10.1371/journal.pone.0340089 (PMC12829834; doi:10.1371/journal.pone.0340089)
Supplement: S2 Table — (PDF) [file pone.0340089.s002.pdf]

S2 Table. Pre and post-seminar scores for each sub-component of the CC-GRAS score

Sub-component of CC-GRAS score	Pre-seminar scores			Post seminar scores			P value
	Mean	SD	N	Mean	SD	N	
I can easily imagine how people feel when they are from a different cultural / religious background	3.57	0.931	516	3.91	0.797	288	<0.001
I am able to question my own views	4.18	0.763	515	4.35	0.671	288	0.004
I am aware of how my views are culturally influenced	3.84	0.880	514	4.31	0.720	286	<0.001
I recognise the influence of stereotypes about service users on my thoughts	3.89	0.917	514	4.31	0.778	288	<0.001
I recognise the influence of stereotypes about service users on my behaviour	3.84	0.946	515	4.23	0.783	287	<0.001
I can easily empathise with the situation of other people	4.22	0.727	516	4.39	0.685	287	0.001
I do not like to question my own views	2.25	1.056	513	2.41	1.266	286	0.221
I am aware of how culture shapes individual’s behaviour and thinking	4.08	0.745	511	4.34	0.721	286	<0.001
I am aware of the social context of certain ethnic minority groups	3.54	0.913	514	4.07	0.748	288	<0.001
I can adapt flexibly and creatively to a new situation	3.90	0.787	516	4.22	0.677	286	<0.001

CC-GRAS - Cultural Competence Groningen Reflection Ability Scale
