# Supplementary material for: Embedding trauma-informed, culturally sensitive, and compassionate care training in health and social care curricula: Evaluation of a multidisciplinary student intervention utilizing a pre- post-test survey design
Source: PLoS One. 2026 Jan 23;21(1):e0340089. doi: 10.1371/journal.pone.0340089 (PMC12829834; doi:10.1371/journal.pone.0340089)
Supplement: S3 Table — (PDF) [file pone.0340089.s003.pdf]

S3 Table. Additional quotations

Theme / subtheme	Quotation
**Theme: Enhanced knowledge**	
Awareness of complexity	*"All we learned today about Trauma Awareness was brilliant."* [Female, White Other, Adult Nursing; line 83]<br>*"Never to assume you have an idea of what it is like to be the service user because every experience of trauma is different for each individual." [Female, White Other, Physiotherapy; line 150]*<br>*"I have learnt a lot about trauma aware care and how it affects people in different ways."* [Female, White British, Adult Nursing; line 163]<br>*"Not everyone is the same and there are different types of trauma in staff and patients*." [Female, White British, Child Nursing; line 105]<br>*"Always think about what might be happening with that person that you might not know."* [Female, White British, Paramedic Science; line 119]<br>*"Looking for signs in certain situations where people may be feeling uncomfortable. What you are saying or talking about may be a trigger of trauma to someone so you should be aware of this so to not retraumatize." [Female, White British, Midwifery; line 101]*<br>*"Be aware of the complexities of individuals including patients, colleagues & the public."* [Male, White British, Physiotherapy; line 159]<br>*"Anyone can experience trauma but there are certain factors that might indicate they may have experience it or have been more likely to experience*." [Female, White British, Child Nursing; line 63]<br>*"That trauma can come from many factors such as war and relationships and many more, and that different people present different signs of trauma and understanding these signs will help the patient."* [Male, Asian, Diagnostic Radiography; line 145]<br>*"Being more trauma aware and not presuming you know why someone is acting the way they are."* [Female, White British, Midwifery; line 108]<br>*"People who suffer trauma are not victims but survivors."* [Female, Black, Adult Nursing; line 114]<br>*"In trauma awareness: never pity! Focus on the strength and resilience of the individual having experienced it."* [Female, White Other, Child Nursing; line 132]

Deeper understanding of cultural diversity	*"During placement I will meet people from many varying ethnicities, cultures, faiths and social backgrounds." [Gender not reported, White British, Radiotherapy and Oncology; line 58]*<br><br>*"That cultures vary and I need to support the patient holistically including religion and personal values."* [Female, White British, Adult Nursing; line 164]<br><br>*"More aware of surroundings and cultural views."* [Female, White British, Adult Nursing; line 156]<br><br>*"Healthcare professionals should not be judgmental and should be aware of cultural differences."* [Female, Black, Adult Nursing; line 90]<br><br>*"Be more open to other religious beliefs and do not generalize them."* [Female, Mixed ethnicity, Midwifery; line 194]
Power of words and communication	*"Really listen in order to provide person centered care."* [Female, White British, Midwifery; line 128]<br><br>*"Learning to listen more and talk less." [Gender not reported, Black, Operating Department Practice; line 97]*<br><br>*"Communication is vital, especially when individuals are in situations where they want to be communicated towards nicely and with respect, rather than you trying to rush them and not listening to their opinions."* [Female, Asian, Child Nursing; line 174]<br><br>*"Sensitive communication, ask open questions & remember Acknowledging /Validation /Empathy /Compassion." [Female, White British, Child Nursing; line 161]*<br><br>*"The ability to take the lead in providing evidence based, compassionate and safe nursing interventions requires effective communication skills."* [Gender not reported, Black, Mental Health Nursing; line 162]<br><br>*"To be mindful of how I communicate."* [Female, Black, Adult Nursing; line 70]<br><br>*"Listening carefully to what others say and how they say things."* [Female, White British, Operating Department Practice; line 99]<br><br>*"A better understanding of … body language/non-verbal communication."* [Female, White British; Radiotherapy and Oncology; line 3]

Power of words and communication cont.	*"Active listening is key to build any kind of therapeutic relationship to ensure they feel safe and comfortable to discuss difficult situations."* [Female, White British, Adult Nursing; line 6]<br><br>*"Remember to slooooow down when speaking to persons where English is a second+ language (even though I'm bilingual myself I still need to remember to slow down and simplify my language in interaction with patients and carers as appropriate)."* [Female, White Other, Child Nursing; line 132]
Position, privilege and unconscious bias	*"The power wheel was very interesting as it showed I … have more power than I realized." [Female, White British, Social Work; line 154]*<br><br>*"I have a wider range of knowledge on my own powers/privilege, but as well as that I can also understand how others may feel when they fall out of the norm of those powers." [Female, Asian, Adult Nursing; line 80]*<br><br>*"How much I am in a perceived position of power and privilege, and to be aware of unconscious bias." [Male, White British, Adult Nursing; line 218]*<br><br>*"A lack of power or perceived marginalization can impact upon an individual." [Female, Black, Occupational Therapist; line 217]*<br><br>*"Unconscious biases , you have to be socially aware of when they might come into play and try hard to stay away from them." [Male, Asian, Paramedic; line 19]*<br><br>*"How easily we stereotype without being aware." [Female, White British, Adult Nursing; line 86]*<br><br>*"It's crucial to appreciate the efforts of multidisciplinary teams and how easily stereotyping can encroach upon the safe effective delivery of person centered care and building trust within relationships with service users." [Female, White British; Child Nursing; line 127]*<br><br>*"Recognizing your own bias is uncomfortable but necessary." [Gender not reported, White British, Midwifery; line 52]*<br><br>*"To be aware of your internal bias/judgements but to make sure this does not impact on the care that you give." [Female, White British, Midwifery; line 101]*

Value of holistic and compassionate care	*“How you treat others can positively or negatively affect their treatment regardless of whether the "medical" issue is solved.”* [Gender not reported, White British, Midwifery; line 52]<br>*“How important it is in adapting care for patients, who may be all struggling with the same thing but are all different individuals with different beliefs and past lives. To ask them what they would like rather than just following a script to fix the problem.”* [Female, White British, Adult Nursing; line 68]<br>*“That the service user wants to be listened to and treated as an individual and not as other patients, their needs must be adapted to suit them.”* [Female, White British, Mental Health Nursing; line 82]<br>*“Everyone is individual with different lives experiences. Person centered care should be the forefront of our service.”* [Female, White British, Midwifery; line 106]<br>*“We have to be aware of a person’s biopsychosocial picture to provide holistic person centered care.”* [Male, White British, Physiotherapy; line 159]<br>*“How to be holistic in my approaches to individuals, understanding everyone is their own person and each have specific wants and needs.”* [Female, White British, Child Nursing; line 62]<br>*“Having empathy and being compassionate is important.”* [Female, Asian, Radiotherapy and Oncology, line 84]<br>*“Being compassionate and recognizing that everyone has their own individual needs.”* [Female; Asian, Midwifery; line 23]
**Theme: Contextual influences**	
Learning by experience and exposure	*“Confidence. Speaking up and challenging things when they are wrong (as I am a student, my views are limited in the clinical world).” [Male, Black, Radiotherapy and Oncology; line 135]*<br>*When responding to what might get in the way of putting into practice what they had learnt: “My knowledge and others' acceptance of me.” [Female, White British, Learning Disability Nurse; line 205]*<br>*“Critical thinking and self-reflection are significant in developing oneself against adopting distancing measures against distress in an emotionally overwhelming clinical context which in turn can affect the efficient effective delivery of person centered care. I learnt so much.”* [Female, White British, Child Nursing; line 127]

Organizational culture	*"Practice supervisors not always recognizing their bias."* [Female, White British, Midwifery; line 128]<br>*"Social culture in a ward."* [Male, White British, Adult Nursing; line 133]<br>*"Authoritative structures in the hospitals – management."* [Male, White British, Diagnostic Radiography; line 87]<br>*"Resistance to change, lack of opportunity due to other staff taking the lead."* [Gender not reported, White British, Radiotherapy and Oncology; line 58]<br>*"Time and pressures of the working environment."* [Female, White British, Physiotherapy; line 98]
Self-care	*"Myself not being at my full functioning mental state, or if I am tired etc. because when communicating I should be reflecting during the episode of care, thinking about my language, am I being respectful? Am I being biased without realizing? Reflecting in action requires me to be mindful and alert. If I am not feeling 100% or not in the right state of mind, I am less likely to be doing this."* [Female, Mixed ethnicity, Adult Nursing; line 208]<br>*"Self-care is important and not to burn out. Reminder to filter and not fall into the pit of vicarious trauma."* [Female, Asian, Physiotherapy; line 141]<br>*"Knowing to look after my mental health so I can provide the best service possible."* [Female, White Other, Diagnostic Radiography; line 91]<br>*"Take care of yourself."* [Female, Black, Adult Nursing; line 14]<br>*"The stress bucket and self-care strategies."* [Female, White British, Social Work; line 186]
**Theme: Reflection and implementation in practice**	
About the course	*"It was food for thought" [Female, White Other, Adult Nursing; line 11]*<br>*"It is an eye-opener; I am aware of how biased one can be when dealing with service users." [Female, Black, Adult Nursing; line 153]*<br>*"Really enjoyed it, learnt a lot." [Female, White British, Midwifery; line 128]*<br>*"Today's session is extremely vital as, I will be working with people that have being traumatized, I will work with people from different cultures, therefore knowing how to support them professionally is extremely import." [Male, Black, Diagnostic Radiography; line 4]*
